# Prevalence of sepsis among adults in China: A systematic review and meta-analysis

**DOI:** 10.3389/fpubh.2022.977094

**Published:** 2022-10-11

**Authors:** Siyuan Lei, Xuanlin Li, Hulei Zhao, Yang Xie, Jiansheng Li

**Affiliations:** ^1^Co-Construction Collaborative Innovation Center for Chinese Medicine and Respiratory Diseases by Henan & Education Ministry of P.R. China, Henan University of Chinese Medicine, Zhengzhou, China; ^2^College of Basic Medical Science, Institute of Basic Research in Clinical Medicine, Zhejiang Chinese Medical University, Hangzhou, China; ^3^Department of Respiratory Diseases, The First Affiliated Hospital of Henan University of Chinese Medicine, Zhengzhou, China

**Keywords:** sepsis, China, prevalence, systematic review, meta-analysis

## Abstract

**Background:**

Sepsis is a major public health problem that cannot be ignored in China and even in the world. However, the prevalence of sepsis in Chinese adults varies among different studies.

**Objective:**

To evaluate the prevalence of hospital-wide sepsis and intensive care unit (ICU) sepsis in Chinese adults.

**Methods:**

PubMed, EMBASE, Cochrane Library, Web of science, China National Knowledge Infrastructure, Chinese biomedical literature service system, Wanfang Database, and VIP databases were systematically searched for studies on sepsis in China published before March 2, 2022. Random effects model was used to calculate pooled prevalence estimates with 95% confidence interval. Subgroup and sensitivity analyses were performed to address heterogeneity. Funnel plots and Egger's test were used to assess the publication bias.

**Results:**

Overall, nine observational studies involving 324,020 Chinese patients (9,587 patients with sepsis) were analyzed. Four hospital-wide studies involving 301,272 patients showed pooled prevalence and mortality of 3.8% (95% CI: 2.9–4.7%, *I*^2^ = 99.9%) and 26% (95% CI: 16–36%, *I*^2^ = 98.0%), respectively. Five studies of ICU sepsis involving 22,748 patients presented pooled prevalence and mortality of 25.5% (95% CI: 13.9–37.0%, *I*^2^ = 99.8%) and 40% (95%CI: 34–47%, *I*^2^ = 95.9%), respectively. Subgroup analysis of sepsis in ICUs revealed that the pooled prevalence was higher among males [17% (95% CI 9–24%, *I*^2^ = 99.6%)], in lung infections [66% (95%CI: 54–77%, *I*^2^ = 98.7%)], and Gram-negative bacteria infections [37% (95%CI: 26–47%, *I*^2^ = 98.3%)]. The pooled prevalence of sepsis, severe sepsis and septic shock was 25.5% (95%CI: 13.9–37.0%, *I*^2^ = 99.8%), 19% (95%CI: 9–28%, *I*^2^ = 99.6%), and 13% (95%CI: 7–19%, *I*^2^ = 99.2%), respectively.

**Conclusions:**

Sepsis is prevalent in 25.5% of ICU patients in China, and sex, sepsis severity, infection site, causative microorganism, and infection type are significant influencing factors. Larger trials are needed to evaluate the prevalence of sepsis in China, which may help the development of global strategies for sepsis management.

**Systematic review registration:**

PROSPERO, identifier: CRD42022314274.

## Introduction

Sepsis is a life-threatening organ dysfunction caused by a dysregulated host response to infection (1), which has become a leading cause of mortality and critical illness worldwide (2). Approximately 48.9 million cases of sepsis and 11 million sepsis-related deaths were recently reported, accounting for 19.7% of all global deaths (3). Sepsis is a major global public health concern characterized by high morbidity, high mortality, and heavy economic burden (4). Sepsis survivors often have long-term physical (5), psychological (6), and cognitive impairments (7) with significant health care and social implications. Thus, improving the prevention, recognition, and treatment of sepsis was declared as a global health priority by the World Health Organization (WHO) in 2017 (8). Sepsis is considered as the most expensive disease of hospitals in the United States, costing approximately $23.7 billion annually and accounting for 6.2% of the aggregate costs for all hospitalizations, which may be higher in low and middle-income countries (9, 10).

Several systematic reviews and meta-analyses have reported the global prevalence of sepsis (11–13). Fleischmann *et al* pooled data on sepsis from 1979 to 2015 across seven high-income countries and reported a population incidence rate of 288 hospital-treated sepsis cases and 148 severe sepsis cases per 100000 person-years (11). However, due to limited data, the incidence of sepsis in low and middle-income countries was not estimated. Markwart et al. assessed the epidemiology of sepsis acquired in hospitals and intensive care units (ICUs) separately and revealed that the pooled incidence of hospital-acquired sepsis was 15.4 cases per 1000 patients and that of ICU acquired sepsis was 44.8 cases per 1000 ICU patients (12). In a recent meta-analysis, the pooled incidence of hospital-treated sepsis was 189 per 100000 person-years, of which 26.7% of sepsis patients died, while the incidence of ICU-treated sepsis was 58 per 10,0000 person-years, of which 41.9% of patients died prior to hospital discharge (13).

In China, sepsis is a great challenge due to the progressive aggravation of the aging population. Recent research has focused on risk factors, mechanisms, and treatments for sepsis (14–16), but the management of epidemiology is also important. Although several studies (17–25) have reported the prevalence of sepsis in China, the prevalence of sepsis between studies was inconsistent, possibly owing to differences in the survey periods, sampling, study sites, diagnostic criteria, and sample demographic characteristics. As the most populous and largest developing country globally, the epidemiological status of sepsis in China cannot be ignored. However, so far there has been no independent systematic review and meta-analysis on the prevalence of sepsis in China. With the rapid development of critical care medicine in the past years, having a deeper insight into the epidemiologic patterns of sepsis in Chinese adults contributes to better managing this disease and providing an evidence-based basis for governments to make relevant public health strategic decisions. In addition, it also has important implications for estimating the burden of sepsis worldwide. Therefore, this review aimed to evaluate the prevalence and characteristics of hospital-wide and ICU sepsis in China.

## Materials and methods

This systematic review and meta-analysis was performed according to the guidelines in the Preferred Reporting Items for Systematic Reviews and Meta-Analyses statement (PRISMA 2020), and the protocol was submitted to the Prospective Register for Systematic Reviews (PROSPERO) (CRD42022314274).

### Search strategy

Two researchers independently and systematically searched eight different databases, including PubMed, EMBASE, Cochrane Library, Web of science, China National Knowledge Infrastructure, Chinese biomedical literature service system, Wanfang Database, and VIP database from their inception to March 2, 2022. The Medical Subject Headings (MESH) terms and keywords used in the search were as follows: (“sepsis” OR “septicemia^*^” OR “septic shock^*^” OR “severe sepsis^*^” OR “systemic inflammatory response syndrome” OR “SIRS” OR “septic” OR “septicaemic shock”) AND (“epidemic^*^” OR “incidence” OR “Prevalence” OR “Morbidity” OR “occur” OR “screen” OR epidemiolog^*^ OR “demograph^*^” OR “etiolog^*^” OR “rate”) AND (“China” OR “Chinese”). In addition, we screened the references of the identified articles and existing systematic reviews to further identify relevant studies. The complete search strategy is detailed in Supplementary Table 1.

### Inclusion and exclusion criteria

Studies were included if they met all of the following criteria: (1)observational design, including cohort, case-control, or cross-sectional studies; (2) study population aged ≥18 years; (3)sepsis diagnosis in accordance with appropriate criteria (such as the sepsis-1 or sepsis-2 or sepsis-3) or the current relevant guidelines; (4) report on the incidence or prevalence of sepsis and available data related to the defined primary outcomes of the current systematic review; and (5) population-based design and conducted in hospital-wide or ICUs in China. The exclusion criteria were as follows: (1) conference abstracts, study protocols, case studies, reviews; (2) duplicate publications; and (3) incomplete data or no relevant outcome.

### Study selection

Two reviewers independently screened the titles and abstracts to identify relevant articles. The full text of each potentially eligible article was then read to identify studies for analysis. Any disagreement was resolved through a discussion with a third investigator.

### Data extraction

Two reviewers independently extracted data on participant and study characteristics according to the guideline for data extraction for systematic reviews and meta-analysis (26), such as first author, publication years, journal, study setting (hospital-acquired or ICU-acquired), sample size, study period, sepsis type, sepsis diagnostic criteria, mean or median age, patients source, sepsis cases, comorbid conditions, number of deaths, risk factors of mortality, duration of ICU or hospital stay, type of infection, infection site and microorganisms.

### Risk of bias assessment

The quality of included studies was evaluated using the 10-item disease prevalence quality tool modified by Hoy et al. (27). Each study is assigned a total score ranging from 0 to 10, with higher scores indicating better study quality. The study quality was then classified based on these scores as low (0–5), moderate (6–8) or high (8–10), respectively.

### Statistical analysis

We extracted sepsis events and the total sample size to calculate the prevalence of sepsis, number of deaths and sepsis events for each included study to calculate mortality. The random-effects model was used to separately estimate the ICU and hospital-wide prevalence of sepsis. The Chi-square test and *I*^2^ value were implemented to assess heterogeneity, and *P* < 0.1 or *I*^2^≥ 75% was defined as significant heterogeneity. Subgroup analysis was performed to determine whether the prevalence was influenced by sex and severity of sepsis. Sensitivity analyses was conducted by plotting the pooled prevalence and excluding one study each time to judge the robustness of our results. Funnel plots and Egger's test were used to assess publication bias. All statistical analyses were performed using Stata software (version 15.1), and *P* < 0.05 was considered statistically significant.

## Results

### Search results

**Figure 1:**
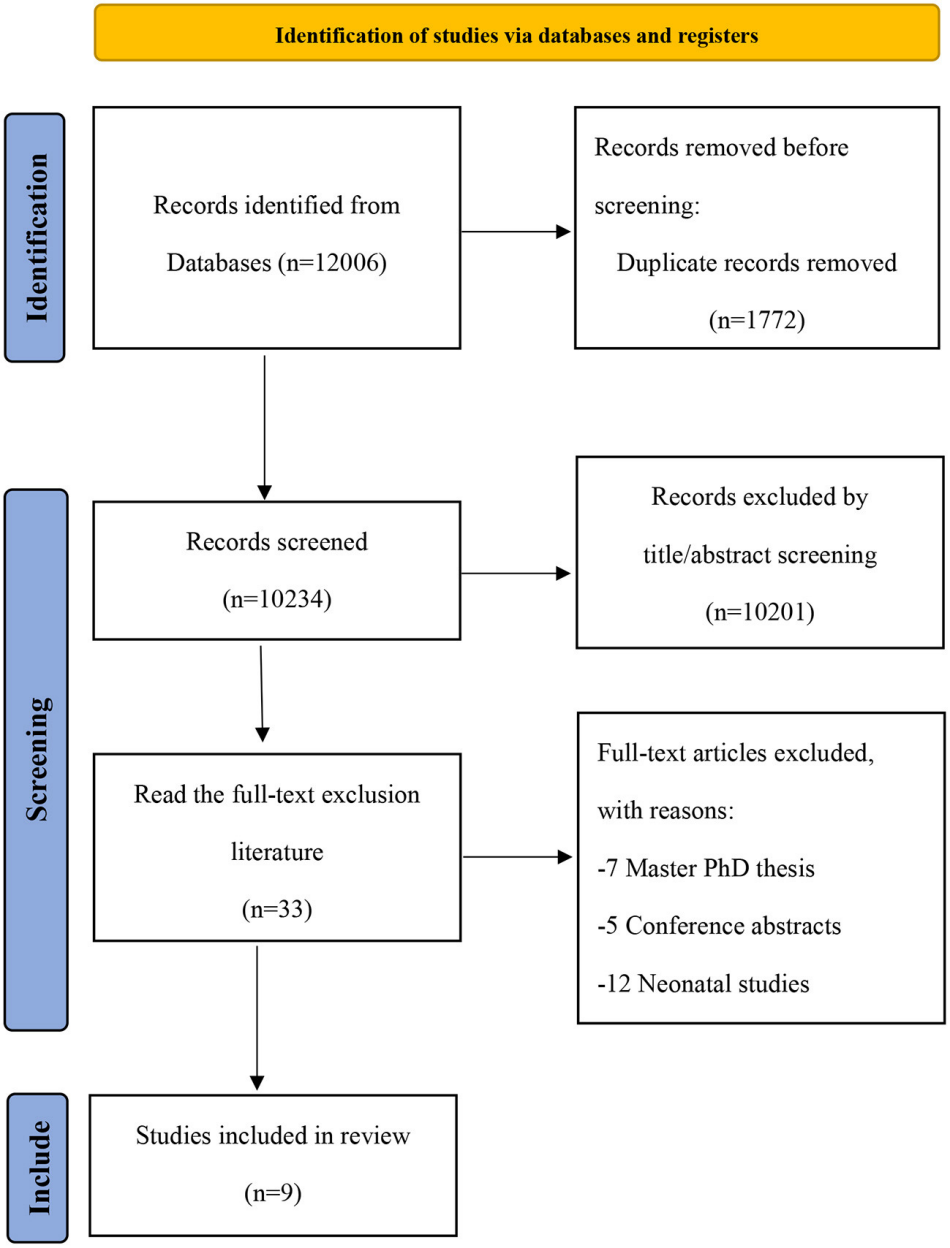
Literature screening flow chart.

A total of 12,006 articles were found, after removing 1,772 duplicates, the titles and abstracts of 10,201 articles were screened, and 33 articles were subjected to a full-text review. Of these, 24 articles were excluded: 7 articles were Master PhD thesis, 5 articles were conference abstracts, and 12 articles were neonatal studies (Supplementary Table 2). Finally, nine observational studies involving 9,587 patients with sepsis were included in the analysis. The search selection process is shown in Figure 1.

### Studies characteristics

The characteristics of the included studies are presented in Table 1. The studies were published between 2000 and 2021, and the sample size ranged from 1,297 to 171,707. Eight studies were published in English-language journals, while one study was in a Chinese-language journal. Of the nine studies, four (17, 20, 21, 25) were conducted in hospital-wide, and five (18, 19, 22–24) were conducted in ICUs. In addition, four (17, 20, 21, 25) studies were retrospective in design, four (18, 19, 22, 24) were prospective, and one (23) was a cross-section survey. The mean age of patients ranged from 36.8 years to 81 years. Sepsis was diagnosed following the Sepsis-1.0/3.0 criteria in three trials (23–25), American College of Chest Physicians/Society of Critical Care Medicine consensus in three studies (18–20), and current relevant guidelines in another three studies (17, 21, 22).

**Table 1:** Characteristics of studies included in the review.

**References**	**Journal**	**Publication** **Language**	**Study type**	**Study period**	**Sample size**	**Sepsis type**	**Sepsis diagnostic criteria**	**Mean or median age**	**Event**	**Male/** **female**
**Hospital patients**										
Xiu et al. (17)	Strait Journal of Preventive Medicine	Chinese	Retrospective cohort study	1992–1997	87183	sepsis	/	36.83	230	152/78
Zhou et al. (20)	Critical Care Medicine	English	Retrospective cohort study	2012.07.01–2014.06.30	21191	Sepsis/severe sepsis/septic shock	American College of Chest Physicians/Society of Critical Care Medicine consensus	80(66–85)	1716	988/728
Jiang et al. (21)	Journal of Critical Care	English	Retrospective study	2013.01–2016.12.	171707	sepsis	(1)Fever (>38 °C), chills, hypotension after a hospital stay of>48 h; (2) The same pathogenic organism was isolated from one or more blood cultures.	67 ± 18	704	431/273
Tian et al. (25)	Chinese Medical Journal	English	Retrospective cohort study	2012.07.01–2014.06.30	21191	Sepsis/severe sepsis/septic shock	Spesis−3.0	81(74–86)	1433	885/548
**ICU patients**										
Cheng et al. (18)	Crit Care Med	English	Prospective observational study	2004.12.1–2005.11.30	3665	severe sepsis	American College of Chest Physicians/Society of Critical Care Medicine Consensus Conference	64(47–74)	318	206/112
Zhou et al. (19)	PLOS ONE	English	Prospective observational cohort study	2009.07.01–2009.08.31	1297	severe sepsis\septic shock	American College of Chest Physicians/Society of Critical Care Medicine consensus conference	66(51–77)	484	336/148
Wang et al. (22)	Frontiers in medicine	English	Prospective cohort study	2014.01.01–2015.08.31	4910	Sepsis/sepsis shock	Surviving Sepsis Campaign (SSC): International Guidelines for the Management of Sepsis and Septic Shock: 2016	62.5 ± 17.8	2086	1362/724
Xie et al. (23)	Critical Care Medicine	English	cross–section survey study	2015.12.1–2016.1.31	11272	Sepsis/severe sepsis/septic shock	sepsis−1	60.8 ± 18.4	2322	1499/823
Cao et al. (24)	Med Sci Monit	English	Prospective Multicenter Study	2017.10.10–2018.01.09	1604	sepsis/sepsis shock	sepsis−3.0	67(55–76)	294	185/109

### Characteristics of patients in ICUs

The five (18, 19, 22–24) studies included 22,748 participants, most of whom were from the surgical, medical ward and emergency department before ICU admission. The mean Acute Physiology and Chronic Health Evaluation II (APACHE II) and Sepsis-related Organ Failure Assessment (SOFA) scores of these patients ranged from 18 to 26 and 7 to 10, respectively. These patients were mainly admitted to the ICU with respiratory, cardiovascular, neurological, gastrointestinal, and acute trauma, which were mostly comorbid with other underlying disease. Most patients diagnosed with sepsis developed acute organ dysfunction, with acute respiratory distress syndrome and acute kidney injury being the most common. The mean duration of ICU stay and hospital stay were 4–8 and 8–22 days, respectively. The characteristics of patients in ICUs are shown in Table 2.

**Table 2:** Characteristics of ICU patients included in the review.

**References**	**Patient source**	**APACHE2**	**SOFA**	**Reason for ICU admission**	**Comorbid conditions**	**Organs with acute dysfunction**	**Death number**	**Risk factors of mortality**	**Duration of ICU stay**	**Duration of hospital stay**
Cheng et al. (18)	Surgical	19(14–25)	9(6–13)	Severe acute pancreatitis 64, intestinal or gastric perforation 62, bowel obstruction 26, infection of liver or gall 33, trauma 59, nosocomial pneumonia 25	Cardiovascular disease 98, respiratory disease 23, gastrointstinal disease 64, malignant neoplasm 38, diabeta 41, organ transplantation 16	Respiratory 290, cardiovascular 131, renal 95, hematologic 123, central nervous system 209, hepatic 146	155(48.7%)	Age, comorbidity of malignant neoplasm, Gram–positive bacteria infection, invasive fungal infection, admission Acute Physiology Score, and admission SOFA scores of respiratory, system and cardiovascular system dysfunction	7(3–14)	22(12–39)
Zhou et al. (19)	Medical371, Scheduled surgery39, Emergency surgery74	21(16–27)	7.5(5–10)	Respiratory disease 259, Gastrointestinal disease 59, Neurological disease 49, Cardiovascular disease 46, Trauma 34, Renal disease 26, Miscellaneous 11	Hypertension 166, diabetes mellitus 85, COPD 80, cancer 55, hematologic malignancy 10, organ transplantation 9, Chronic respiratory failure 73, Chronic heart failure 56, Immunocompromise 48, Chronic renal failure 13, Chronic liver dysfunction 11	AKI 201, ARDS 265	162(33.5%)	APACHE II score, presence of ARDS, bloodstream infection and comorbidity of cancer	7(4–15)	18(10–38)
Wang et al. (22)	/	19.0(14.0–25.0)	7.0(4.0–10.0)	Pneumonia 172, trauma 58, Postoperative monitoring 136, Gastrointestinal 91, Heart failure 89, Neurological 54, other 79	Respiratory disease 332, Cardiovascular disease 390, Hypertension 767, Diabetes mellitus 411, Chronic renal failure 285, Cancer 207, Cirrhosis 38	ARDS 924, AKI870	688(33.0%)	Severity illness scores, length of stay, number of organ dysfunction	8(4.0–16.0)	18.0(10.0–29.0)
Xie et al. (23)	Emergency department 573, Surgical wards992, Medical wards495, Other hospital262,Postoperative sepsis808	18 ± 8.0	7.78 ± 4.1	Respiratory 873, Cardiovascular 134, Digestive/liver 534, Trauma 113, Neurologic 226, Renal 91, Metabolic 21, High risk 182, Other 148	Hypertension 919, Coronary artery disease 395, Heart failure 218, COPD 268, Diabetes mellitus 459, Chronic renal failure 229, Maintenance hemodialysis 87, Solid malignant tumors 286, Hematologic cancer 42, Cirrhosis 72, Connective tissue disease 78, Immunosuppression 178	/	746(32.1%)	Older age, low body weight, higher SOFA score, the number of SIRS criteria, comorbid with heart failure, hematologic cancer, immunosuppression, higher level of lactate, infection site (pneumonia and bloodstream)	8(4–15)	20(10–40)
Cao et al. (24)	Emergency treatment69,Postoperative transfer55, General wards151,Specialty or ICU from other hospitals19	26(21–32)	10(7–12)	Respiratory diseases113, Circulatory diseases10, Diseases of digestive system92, Neurological diseases25, Endocrine system diseases5, Diseases of urinary system22, Hematological diseases4, Rheumatic immune system diseases1, Trauma10,other12	Coronary heart disease45, Diabetes60, Malignant tumors22, Autoimmune diseases11, Chronic renal insufficiency and failure18, Hypertension95, Chronic obstructive pulmonary disease34, Sequelae of cerebrovascular accident18, other65	Respiratory system282, Coagulation system186, liver116, Cardiovascular system200, Central nervous system236, Kidney170	169	acute central nervous system dysfunction, lowest blood phosphorus level, highest lactate level, 24–h APACHE–II score, and lung infection	4(2–8)	8(2–19)

AKI, Acute kidney injury; ARDS, Acute respiratory distress syndrome; COPD, Chronic obstructive pulmonary disease.

### Characteristics of infection and causative organisms in ICU patients

All five (18, 19, 22–24) studies reported the source of infection, site of infection and the microorganism, and sources of infection including ICU acquired, hospital acquired and community-acquired infections. The sites of infection were mainly in the lung, abdomen, intestine, urinary tract, and bloodstream. The common causative microorganisms were Gram-negative bacteria, Gram-positive bacteria, and fungi. Four (18, 19, 22, 24) studies reported pathogenic bacteria, mainly *Staphylococcus aureus, Klebsiella pneumoniae, Pseudomonas aeruginosa, Escherichia coli*, and *Acinetobacter baumannii*. The characteristics of infection and organisms in ICUs are shown in Table 3.

**Table 3:** Characteristics of infection in ICU patients included in the review.

**References**	**Type of infection**	**Infection site**	**Microorganisms**	**Pathogenic bacteria**
Cheng et al. (18)	Documented infection228, ICU–acquired infection135	Abdomen 230, respiratory tract 168, positive blood cultures 90, device related 39, wound surface 54, urinary tract 23, multisite 188	Gram–positive 146, Gram–negative 171, Fungi 90, mixed infection 139	Staphylococcus aureus 40, Klebsiella pneumoniae 27, Pseudomonas aeruginosa 44, Escherichia coli 44, Acinetobacter baumannii 82, Candida tropicalis 27, Candida albicans54, Aspergillus 7
Zhou et al. (19)	ICU–acquired221,non–ICU–acquired48	Pneumonia 419, Intra–abdominal infection 80, Gastroenteritis 41, Urinary tract infection 37, Bloodstream infection 37, Soft tissue infection 34, CNS infection 23, Multiple–site infection 167	Gram positives 39, Gram negatives 168, Fungi 6, mixed infection 49	Staphylococcus aureus 17, Acinetobacter baumannii 38, Klebsiella pneumoniae 25, Pseudomonas aeruginosa 33, Escherichia coli 26, Candida albicans1, Aspergillus4
Wang et al. (22)	Community–acquired1181, Hospital–acquired905	Lung 1157, pleura 121, abdomen 510, urinary tract 88, bloodstream 157, catheter–related sites 42, wound/soft tissue 107, CNS 34, unknown 314	Gram–positive 407, Gram–negative 949, Fungi 326	Staphylococcus 232, Enterococcus 132, Acinetobacter 370, Escherichia 256, Klebsiella 147, Pseudomonas 281, Candida 249, Aspergillus 35
Xie et al. (23)	Community–acquired sepsis1536, Hospital–acquired sepsis750, Healthcare –associated sepsis36	Pneumonia 1581, Bloodstream 181, Abdominal 617, Renal/urinary tract 166, Skin and soft tissue 134, CNS 64, Other139, Unknown 94	Positive isolates 962, Gram–positive 264, Gram–negative 626, Fungi 201, Other organisms 24, Multi–drug resistant organisms 279	/
Cao et al. (24)	Community infection247, Hospital acquired infections47	Lung 191, abdomen 104, urinary system 26, hematological system 18, CNS 13, skin soft tissue 6, others 16	Gram–positive bacteria 48,Gram–negative bacteria 70,Fungus 19	Staphylococcus aureus 11,Enterococcus 7,Klebsiella pneumoniae 25,Escherichia coli 21,Acinetobacter baumannii 8,Pseudomonas aeruginosa 4,Candida 10,Aspergillus 7

CNS, Central nervous system.

### Risk of bias assessment

The quality of the included studies is presented in Table 4. One (17) study with 5 points was judged as low quality, seven (18–22, 24, 25) study with 6–8 points were judged as moderate quality, and one (23) study with 9 was judged as high quality.

**Table 4:** Risk of bias for included studies.

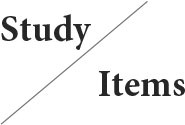	**1**	**2**	**3**	**4**	**5**	**6**	**7**	**8**	**9**	**10**	**Scores**	**Summary risk of bias**
**Hospital–wide studies**												
Xiu et al. (17)	N	N	N	N	Y	N	Y	Y	Y	Y	5	High
Zhou et al. (20)	N	N	N	Y	Y	Y	Y	Y	Y	Y	7	Moderate
Jiang et al. (21)	N	N	N	Y	Y	N	Y	Y	Y	Y	6	Moderate
Tian et al. (25)	N	N	N	Y	N	Y	Y	Y	Y	Y	6	Moderate
**ICU–based studies**												
Cheng et al. (18)	Y	N	N	Y	Y	Y	Y	Y	Y	Y	8	Moderate
Zhou et al. (19)	Y	N	N	Y	Y	Y	Y	Y	N	Y	7	Moderate
Wang et al. (22)	Y	N	N	Y	Y	Y	Y	Y	Y	Y	8	Moderate
Xie et al. (23)	Y	N	Y	Y	Y	Y	Y	Y	Y	Y	9	Low
Cao et al. (24)	N	N	N	Y	Y	Y	Y	Y	Y	Y	7	Moderate

Was the study's target population a close representation of the national population in relation to relevant variables?

Was the sampling frame a true or close representation of the target population?

Was some form of random selection used to select the sample, or was a census undertaken?

Was the likelihood of non-response bias minimal?

Were data collected directly from the subjects (as opposed to a proxy)?

Was an acceptable case definition used in the study?

Was the study instrument that measured the parameter of interest shown to have validity and reliability?

Was the same mode of data collection used for all subjects?

Was the length of the shortest prevalence period for the parameter of interest appropriate?

Were the numerator(s) and denominator(s) for the parameter of interest appropriate?

The summary risk of bias of studies was graded as “high risk” if the risk of bias of two or more items was judged as “high.” The summary risk of bias was graded as “moderate risk” if the risk of bias of one item was judged as “high” and a maximum of one other item was judged as “moderate.” In addition, the overall risk of bias was graded as “moderate” if two or a maximum of three items were judged as “moderate” risk of bias. The summary risk of bias of studies was judged as “low” if the risk of bias of a maximum of one item was judged as “moderate”.

### Prevalence of sepsis in China

Four (17, 20, 21, 25) studies provided data on the prevalence of hospital-wide sepsis and five (18, 19, 22–24) studies provided data on ICU sepsis. The overall pooled prevalence was 3.8% (95%CI: 2.9–4.7%, *I*^2^ = 99.9%, *P* = 0.000) for hospital-wide sepsis and 25.5% (95%CI: 13.9–37.0%, *I*^2^ = 99.8%, *P* = 0.000) for ICU sepsis (Tables 5, 6).

**Table 5:** The prevalence of sepsis in hospital–wide and ICUs in China.

**Setting**	**References**	**Prevalence**	**95%CI**	**Weight (%)**
**Hospital–wide**	Xiu et al. (17)	0.3%	0.2–0.3%	25.48
	Zhou et al. (20)	8.1%	7.7–8.5%	24.44
	Jiang et al. (21)	0.4%	0.4–0.4%	25.48
	Tian et al. (25)	6.8%	6.4–7.1%	24.60
	Overall	3.8%	2.9–4.7%	100.00
**ICUs**	Cheng et al. (18)	8.7%	7.8–9.6%	20.06
	Zhou et al. (19)	37.3%	34.7–39.9%	19.88
	Wang et al. (22)	42.5%	41.1–43.9%	20.03
	Xie et al. (23)	20.6%	19.9–21.3%	20.07
	Cao (24)	18.3%	16.4–20.2%	19.98
	Overall	25.5%	13.9–37.0%	100.00

**Table 6:** The mortality of sepsis in hospital–wide and ICUs in China.

**Setting**	**References**	**Mortality**	**95%CI**	**Weight (%)**
**Hospital–wide**	Xiu et al. (17)	19%	14–24%	24.14
	Zhou et al. (20)	21%	19–22%	25.48
	Jiang et al. (21)	24%	21–28%	25.07
	Tian et al. (25)	39%	37–42%	25.30
	Overall	26%	16–36%	100.00
**ICUs**	Cheng et al. (18)	49%	43–54%	18.94
	Zhou et al. (19)	33%	29–38%	19.93
	Wang et al. (22)	33%	31–35%	21.13
	Xie et al. (23)	32%	30–34%	21.18
	Cao et al. (24)	57%	52–63%	18.81
	Overall	40%	13.9–37.0%	100.00

### Hospital mortality of sepsis

All nine (17–25) studies provided the number of sepsis deaths. The estimated pooled of hospital mortality of sepsis was 26% (95%CI: 16–36%, *I*^2^ = 98.0%, *P* = 0.000) for hospital-wide and 40% (95%CI: 34–47%, *I*^2^ = 95.9%, *P* = 0.000) for ICU sepsis (Tables 5, 6).

### Subgroup analysis of ICU sepsis

A separate meta-analysis was performed using random effects models for subgroup effects of sex, sepsis severity, site of infection, microorganisms, and type of infection on ICU sepsis. The pooled prevalence of ICU sepsis was 17% (95% CI 9–24%, *I*^2^ = 99.6%, *P* = 0.000) among males, and 9% (95% CI 5–12%, *I*^2^ = 99.1%, *P* = 0.000) for females (Table 7).

**Table 7:** Subgroup analysis of the prevalence of ICUs sepsis.

**Subgroups**	**Number** **of studies**	**Model**	**Pooled estimate**	**Heterogeneity**	
			**(95%CI)**	**I^2^**	***P***
**Gender**					
Male	5	Random	0.17(0.09, 0.24)	99.6%	0.000
Female	5	Random	0.09(0.05, 0.12)	99.1%	0.000
**Sepsis severity**					
Sepsis	5	Random	0.255(0.139, 0.370)	99.8%	0.000
Severe sepsis	3	Random	0.19(0.09, 0.28)	99.6%	0.000
Septic shock	4	Random	0.13(0.07, 0.19)	99.2%	0.000
**Infection site**					
Lung	5	Random	0.66(0.54, 0.77)	98.7%	0.000
Abdomen	5	Random	0.37(0.25, 0.48)	98.8%	0.000
Hematology	5	Random	0.10(0.07, 0.14)	94.1%	0.000
Urinary Tract	5	Random	0.07(0.05, 0.09)	84.6%	0.000
Skin and soft tissue	5	Random	0.07(0.04, 0.09)	91.9%	0.000
Neurology	4	Random	0.03(0.02, 0.04)	82.5%	0.000
**Microorganisms**					
Gram–negative	5	Random	0.37(0.26, 0.47)	98.3%	0.000
Gram–positive	5	Random	0.20(0.12, 0.27)	98.1%	0.000
Fungi	5	Random	0.12(0.05, 0.18)	98.8%	0.000
**Infection type**					
Community–acquired	3	Random	0.69(0.57, 0.81)	98.6%	0.000
Hospital–acquired	5	Random	0.36(0.27, 0.45)	97.7%	0.000

With respect to the severity of sepsis, the pooled prevalence of sepsis, severe sepsis, and septic shock in ICUs were 25.5% (95%CI: 13.9–37.0%, *I*^2^ = 99.8%, *P* = 0.000), 19% (95%CI: 9–28%, *I*^2^ = 99.6%, *P* = 0.000), and 13% (95%CI: 7–19%, *I*^2^ = 99.2%, *P* = 0.000), respectively (Table 7).

According to the site of infection, the pooled prevalence of ICU sepsis was the highest in pulmonary infections at 66% (95%CI: 54–77%, *I*^2^ = 98.7%, *P* = 0.000), followed by that in abdominal infections at 37% (95%CI: 25–48%, *I*^2^ = 98.8%, *P* = 0.000), hematologic infections at 10% (95%CI: 7–14%, *I*^2^ = 94.1%, *P* = 0.000), urinary tract infections at 7% (95%CI: 5–9%, I^2^ = 84.6%, *P* = 0.000), skin and soft tissue infections at 7% (95%CI: 4–9%, *I*^2^ = 91.9%, *P* = 0.000), and the lowest in neurology infections at 3% (95%CI: 2–4%, *I*^2^ = 82.5%, *P* = 0.001) (Table 7).

For the infected microorganisms, ICU sepsis was prevalent in 37% (95%CI: 26–47%, *I*^2^ = 98.3%, *P* = 0.000) of Gram–negative infections, 20% (95%CI: 12–27%, *I*^2^ = 98.1%, *P* = 0.000) of Gram–positive infections, and 12% (95%CI: 5–18%, *I*^2^ = 98.8%, *P* = 0.000) of fungi infections (Table 7).

In terms of the infection type, ICU sepsis was prevalent in 69% (95%CI: 57–81%, *I*^2^ = 98.6%, *P* = 0.000) of community–acquired infections and in 36% (95%CI: 27–45%, *I*^2^ = 97.7%, *P* = 0.000) of hospital–acquired infections (Table 7).

### Sensitivity analyses

The sensitivity analyses showed that no individual study altered the pooled results, indicating that the pooled prevalence for ICU sepsis, hospital mortality for hospital–wide sepsis and ICU sepsis has good stability. However, there was large heterogeneity in the prevalence of hospital–wide sepsis (Supplementary Table 3).

### Publication bias

**Figure 2:**
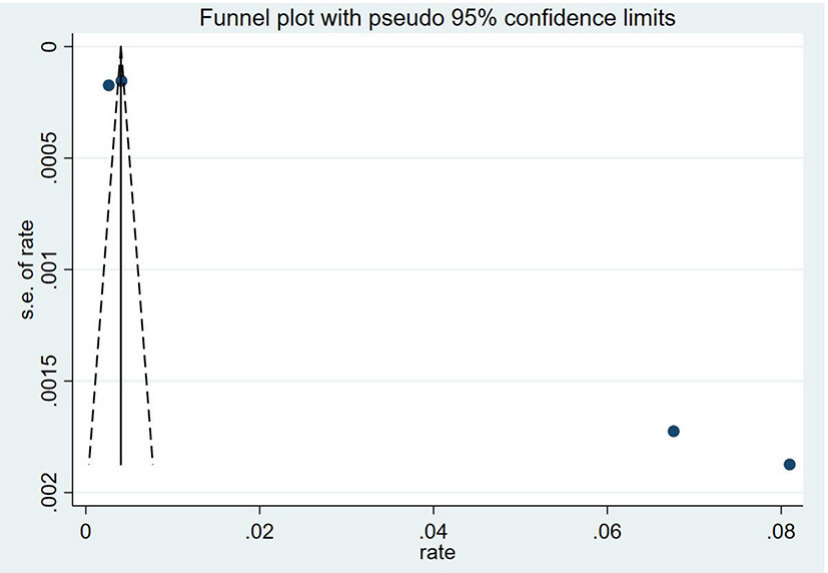
Funnel figure showing the prevalence of hospital–wide sepsis.

**Figure 3:**
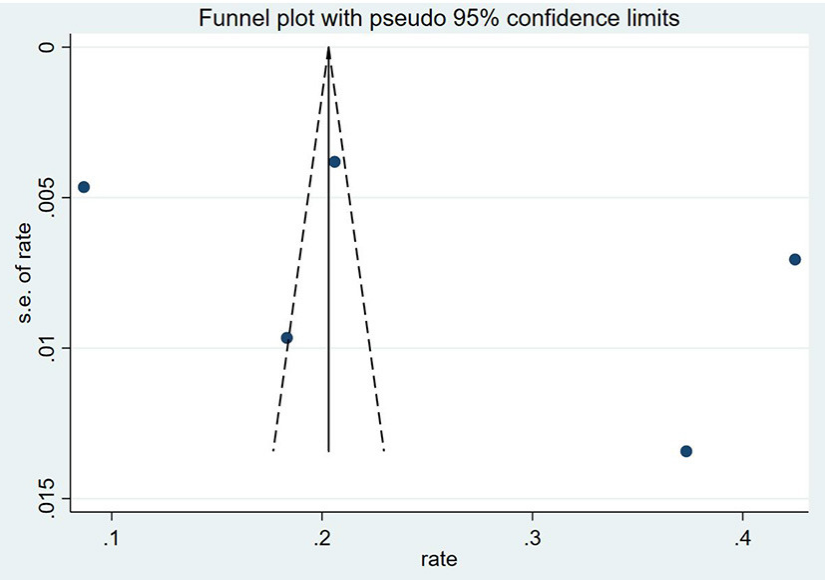
Funnel figure showing the prevalence of ICUs sepsis.

The funnel plots and the Egger's test demonstrated that there was no publication bias on the prevalence for ICU sepsis (*P* = 0.452). The result of Egger's test was *P* = 0.017. Meanwhile, the asymmetric funnel plot showed that publication bias existed among the studies on the prevalence for hospital–wide sepsis (Figures 2, 3).

## Discussion

In this systematic review of studies from China, the pooled prevalence was 3.8% (95%CI: 2.9–4.7%) for hospital–wide sepsis and 25.5% (95%CI: 13.9–37.0%) for ICU sepsis. The estimated pooled of hospital mortality for hospital–wide sepsis and ICU sepsis was 26% (95%CI: 16–36%) and 40% (95%CI: 34–47%), respectively. In addition, sex, sepsis severity, infection site, causative microorganisms and infection type were identified as significant factors of prevalence of ICU sepsis. To the best of our knowledge, this is the first meta–analysis to examine the prevalence of sepsis in China. Our findings could provide strong evidence about high prevalence of sepsis in China.

Sepsis could develop from healthcare–associated infections, and the WHO has provided strong evidence and recommendations on the effectiveness of infection prevention and control measures to reduce the incidence of hospital–wide sepsis (28). A 2011 global report by the WHO showed that the prevalence of hospital–wide sepsis ranges 5.7 to 19.1% (29), which indicates that sepsis still deserves our attention in hospital–wide. Our results were higher than those in the recent meta–analysis by Markwart et al. (12), which may be because most of the studies they included were from high–income countries and because sepsis has attracted great attention and active prevention and treatment in various countries in recent years. However, further improvements are needed considering that only 28% of countries worldwide have implemented functional measures, most of which are developed countries (30). Moreover, there may be objective reasons for the high prevalence in China, and these primarily could be due to the aging population, the large number of elderlies with comorbidities, and higher rates of antimicrobial resistance, which may lead to higher rates of hospital–wide sepsis.

Sepsis also has a more crucial impact in ICUs, and there have been several studies on the prevalence of ICU sepsis. A study analyzed more than 17,000 sepsis cases in the USA and found that 55% of these cases required ICU admission (31). In a Brazilian study, which included 317 ICUs, the incidence and mortality of ICU sepsis in adult was high at 30.2 and 55.7%, respectively (32). However, a prospective, multicenter, observational study in Italy conducted by Sakr et al. (33) indicated that the incidence of ICU sepsis was only 11.4%, and the ICU mortality was 41.3%, with the mortality rate increased with the severity of sepsis. In the current study the prevalence and mortality of ICU sepsis in China was 25.5 and 40%, lower than those reported in Brazil (32) but higher than those reported in Italy (33). These data suggest that the prevalence of ICU sepsis varies widely among countries, and the difference may be related to the higher burden of infectious diseases, varying patterns of underlying comorbidities, limited infection prevention strategies, and fewer resources for sepsis treatment and intensive care in low and middle–income countries. In addition, it may also be related to the large variation in development among regions in China and the insufficient attention paid by physicians to sepsis, leading to delayed treatment.

Subgroup analysis showed that the prevalence of ICU sepsis was higher in males than in females, consistent with the results of a German study (34). This may be related to the fact that comorbidities are more common in male (35). However, due to limited information, further studies are warranted to explore this phenomenon. We also found that the prevalence of ICU sepsis was negatively correlated with sepsis severity, which is similar to a previous finding (36) that sepsis is more prevalent than severe sepsis, and septic shock is the least prevalent.

As in other epidemiologic studies of sepsis (37–39), the most common site of infection in our study was the lung, followed by the abdomen. This finding indicated when the source of infection remains unknown in a sepsis patient in the ICU, clinicians should preferentially consider pulmonary and abdominal sources. Furthermore, it also suggested the importance of implementing effective strategies to prevent both community–acquired and hospital–acquired pneumonia. The analysis of microbiological characteristics of ICU sepsis in our study showed that Gram–negative bacteria were the most common pathogens, consistent with other studies (40, 41). However, several studies in developed countries reported a predominance of Gram–positive bacteria (42, 43). These differences may be explained by differences in underlying conditions, geographical variations, case mix, antibiotic prescription habits, invasive interventions, and the quality of care provided. Moreover, there might be bias in the results of microorganisms as not all studies reported microbiological findings. In addition, our study found that the majority of infections in ICU sepsis patients were acquired in the community, accounting for 69% of all ICU sepsis, in line with the results by Rudd et al. (3).

The strengths of this study are that it provided a comprehensive review of the prevalence of sepsis both hospital–wide and in ICUs in China and included a large number of studies, either in English or in Chinese. Second, we performed a pooled analysis of the characteristics of ICU sepsis according to sex, severity, microorganism, and site of infection. However, we also acknowledge several limitations in our study. Firstly, the definitions of sepsis were heterogeneous, and thus, the estimation of the prevalence might be biased. Second, our study did not cover all provinces and regions in China, restricting the generalizability of the findings. Differences in the prevalence of sepsis across regions could not be explored because of limited information in the included studies. Third, significant heterogeneity exists in our review. Despite our effort, the source of heterogeneity could not be identified because of the limited information in the primary studies. Lastly, important factors related to sepsis mortality, such as age, comorbidities, microbial distribution and use of medicine, were not analyzed due to insufficient data. More high–quality studies are required to report on the epidemiology of sepsis and resolve the many unknown factors related to sepsis.

## Conclusions

This systematic review shows that the pooled prevalence of hospital–wide and ICU sepsis remains high in China. These findings provide evidence–based data that can help promote further sepsis research and prevention efforts throughout the nation, and contribute to develop global strategies for sepsis.

## Data availability statement

The original contributions presented in the study are included in the article/Supplementary material, further inquiries can be directed to the corresponding author.

## Author contributions

JL and XL would answer for design and conception of the article. SL, XL, and HZ would answer for the collection, assembly of materials and would answer for data interpretation, analysis. SL and XL drafted the manuscript and contributed equally to the manuscript. XL, HZ, YX, and JL revised it. All authors reviewed and approved the final version of the manuscript.

## Funding

The author(s) disclosed receipt of the following financial support for the research, authorship, and/or publication of this article: the Chinese Medicine Inheritance and Innovation Hundred and Ten Million Talent Project—Chief Scientist of Qi–Huang Project ([2020] No. 219); Zhong–yuan Scholars and Scientists Project (No.2018204); and Characteristic backbone discipline construction project of Henan Province (STG–ZYX03–202123).

## Conflict of interest

The authors declare that the research was conducted in the absence of any commercial or financial relationships that could be construed as a potential conflict of interest.

## Publisher's note

All claims expressed in this article are solely those of the authors and do not necessarily represent those of their affiliated organizations, or those of the publisher, the editors and the reviewers. Any product that may be evaluated in this article, or claim that may be made by its manufacturer, is not guaranteed or endorsed by the publisher.
