# Supplementary material for: Prevalence of sepsis among adults in China: A systematic review and meta-analysis
Source: Front Public Health. 2022 Oct 11;10:977094. doi: 10.3389/fpubh.2022.977094 (PMC9596150; doi:10.3389/fpubh.2022.977094)
Supplement: Supplementary file 1 [file Data_Sheet_1.doc]

**Supplementary table 1**

**Details of the Literature Search Strategy**

(1) PubMed

| **Search** | **Query** | **Items found** |
| --- | --- | --- |
| #1 | (("Sepsis"[Mesh]) OR "Shock, Septic"[Mesh]) | 133741 |
| #2 | (((((((Sepsis*[Title/Abstract]) OR (Septicemia*[Title/Abstract])) OR (Septic shock*[Title/Abstract])) OR (Severe sepsis*[Title/Abstract])) OR (Systemic Inflammatory Response Syndrome[Title/Abstract])) OR (SIRS[Title/Abstract])) OR (septic[Title/Abstract])) OR (septicaemic shock[Title/Abstract]) | 165782 |
| #3 | #1OR#2 | 236024 |
| #4 | "Epidemiology"[Mesh] OR "Prevalence"[Mesh] | 351560 |
| #5 | (((((((((epidemic*[Title/Abstract]) OR (incidence[Title/Abstract])) OR (Prevalence[Title/Abstract])) OR (Morbidity[Title/Abstract])) OR (occur[Title/Abstract])) OR (screen[Title/Abstract])) OR (epidemiolog*[Title/Abstract])) OR (demograph*[Title/Abstract])) OR (etiolog*[Title/Abstract])) OR (rate[Title/Abstract]) | 5212557 |
| #6 | #4 OR #5 | 5276566 |
| #7 | "China"[Mesh] | 239678 |
| #8 | "China"[Title/Abstract] OR "Chinese"[Title/Abstract] | 430027 |
| #9 | ("China"[Mesh]) OR ((China[Title/Abstract]) OR (Chinese[Title/Abstract])) | 491021 |
| #10 | #3 AND #6 AND #9 | 1310 |

(2) Embase

| **Search** | **Query** | **Items found** |
| --- | --- | --- |
| #1 | 'sepsis'/exp | 308060 |
| #2 | 'septic shock'/exp | 65018 |
| #3 | sepsis*:ab,ti OR septicemia*:ab,ti OR 'septic shock*':ab,ti OR 'severe sepsis*':ab,ti OR 'systemic inflammatory response syndrome':ab,ti OR sirs:ab,ti OR septic:ab,ti OR 'septicaemic shock':ab,ti | 243663 |
| #4 | #1 OR #2 OR #3 | 384947 |
| #5 | 'prevalence'/exp | 866503 |
| #6 | 'epidemiology'/exp | 3996034 |
| #7 | epidemic*:ab,ti OR incidence:ab,ti OR prevalence:ab,ti OR morbidity:ab,ti OR occur:ab,ti OR screen:ab,ti OR epidemiolog*:ab,ti OR demograph*:ab,ti OR etiolog*:ab,ti OR rate:ab,ti | 6992961 |
| #8 | #5 OR #6 OR #7 | 8763382 |
| #9 | 'china'/exp | 282251 |
| #10 | china:ab,ti OR chinese:ab,ti | 518514 |
| #11 | #9 OR #10 | 592654 |
| #12 | #4 AND #8 AND #11 | 1842 |

(3) Cochrane Library

| **Search** | **Query** | **Items found** |
| --- | --- | --- |
| #1 | MeSH descriptor: [Sepsis] explode all trees | 4821 |
| #2 | MeSH descriptor: [Shock, Septic] explode all trees | 1050 |
| #3 | (Sepsis*):ti,ab,kw OR (Septicemia*):ti,ab,kw OR (Septic shock*):ti,ab,kw OR (Severe sepsis*):ti,ab,kw OR (Systemic Inflammatory Response Syndrome):ti,ab,kw OR (SIRS):ti,ab,kw OR (septic):ti,ab,kw OR (septicaemic shock):ti,ab,kw | 17050 |
| #4 | #1 OR #2 OR #3 | 18616 |
| #5 | MeSH descriptor: [Prevalence] explode all trees | 4906 |
| #6 | MeSH descriptor: [Epidemiology] explode all trees | 42 |
| #7 | (epidemic*):ti,ab,kw OR (incidence):ti,ab,kw OR (Prevalence):ti,ab,kw OR (Morbidity):ti,ab,kw OR (occur):ti,ab,kw OR (epidemic*):ti,ab,kw OR (incidence):ti,ab,kw OR (Prevalence):ti,ab,kw OR (Morbidity):ti,ab,kw OR (occur):ti,ab,kw | 784120 |
| #8 | #5 OR #6 OR #7 | 784121 |
| #9 | MeSH descriptor: [China] explode all trees | 5466 |
| #10 | MeSH descriptor: [China] explode all trees | 40671 |
| #11 | #8 OR #9 | 41134 |
| #12 | #4 AND #8 AND #11 | 289 |

(4) [Web of Science](https://apps.webofknowledge.com/home.do?SID=6BQQjiiMCVa9MgFvRpC) core collection

| **Search** | **Query** | **Items found** |
| --- | --- | --- |
| #1 | Topic: (Sepsis* OR Septicemia* OR "Septic shock*" OR "Severe sepsis*" OR "Systemic Inflammatory Response Syndrome" OR SIRS OR septic OR "septicaemic shock") | 218020 |
| #2 | Topic: (epidemic* OR incidence OR Prevalence OR Morbidity OR occur OR screen OR epidemiolog* OR demograph* OR etiolog* OR rate) | 10754797 |
| #3 | Topic: (China OR Chinese) | 1146034 |
| #4 | #1 AND #2 AND #3 | 1444 |

**Supplemental Table 2 Characteristics of Excluded Studies**

**Characteristics of excluded studies**

| **Author, Year** | **Tittle** | **Reasons** |
| --- | --- | --- |
| Cheng, 2007 | Epidemiology of severe sepsis in intensive care units of ten university affiliated hospitals in China | Master PhD thesis |
| Cheng, 2009 | Epidemiological Study and Copy number Polymorphisms within Defensin Gene Cluster in Severe Sepsis |
| Cui, 2014 | Epidemiologic investigation of sepsis in emergency adults |
| Wang, 2015 | Epidemiology and clinical syndrome of sepsis in 87 patients |
| Tang, 2016 | Epidemiology of sepsis in critical care medicine departments in Western China |
| Sun, 2018 | Epidemiologic features of septic shock: a single center seven-year clinical data analysis |
| Xie, 2019 | Clinical epidemiology and early diagnosis of sepsis in Chinese ICUs |
| French, G. L., 1990 | Septicaemia in Hong Kong | Conference abstracts |
| Yuen, K. Y.,  1990 | Streptococcus zooepidemicus (Lancefield group C) septicaemia in Hong Kong |
| Gan, 2011 | The multicenter study of sepsis in pediatric intensive care units in China |
| Hou, 2018 | Examining epidemiological burden of sepsis in the Chinese population using a focused literature review |
| Liu, 2021 | Epidemiology, risk factors and clinical outcomes of healthcare-associated bloodstream infections due to multidrug-resistant microorganisms in a single-center cohort in northwestern China |
| Chen, 2009 | Clinical epidemiology of severe sepsis in the neonatal intensive care unit | Neonatal studies |
| Collaborative Group for the Study of Sepsis, 2012 | Clinical study on sepsis in 2 pediatric intensive care units in Beijing |
| Tu, 2013 | Investigation of nosocomial sepsis among newborns in a hospital in Wuhan |
| Zhou, 2013 | Survey of the current status of sepsis in children and analysis of risk factors for mortality |
| Wang, 2014 | An epidemiologic survey of pediatric sepsis in regional hospitals in China |
| Luo, 2018 | Epidemiologic investigation of children with community-acquired sepsis |
| Xiao, 2019 | Epidemiology of Pediatric Severe Sepsis in Main PICU Centers in Southwest China |
| Guo, 2019 | A multicenter epidemiological survey of nosocomial infection and sepsis among hospitalized neonates in Shaanxi Province with a gestational age of < 34 weeks and preterm birth |
| Jiang, 2020 | Early-onset sepsis among preterm neonates in China, 2015 to 2018 |
| Jiang, 2020 | Epidemiology and microbiology of late-onset sepsis among preterm infants in China, 2015–2018: A cohort study |
| Pan, 2020 | Late-onset neonatal sepsis in Suzhou, China |
| Cao, 2021 | Assessment of Neonatal Intensive Care Unit Practices, Morbidity, and Mortality among Very Preterm Infants in China |

**Supplemental Table 3 Sensitivity Analysis**


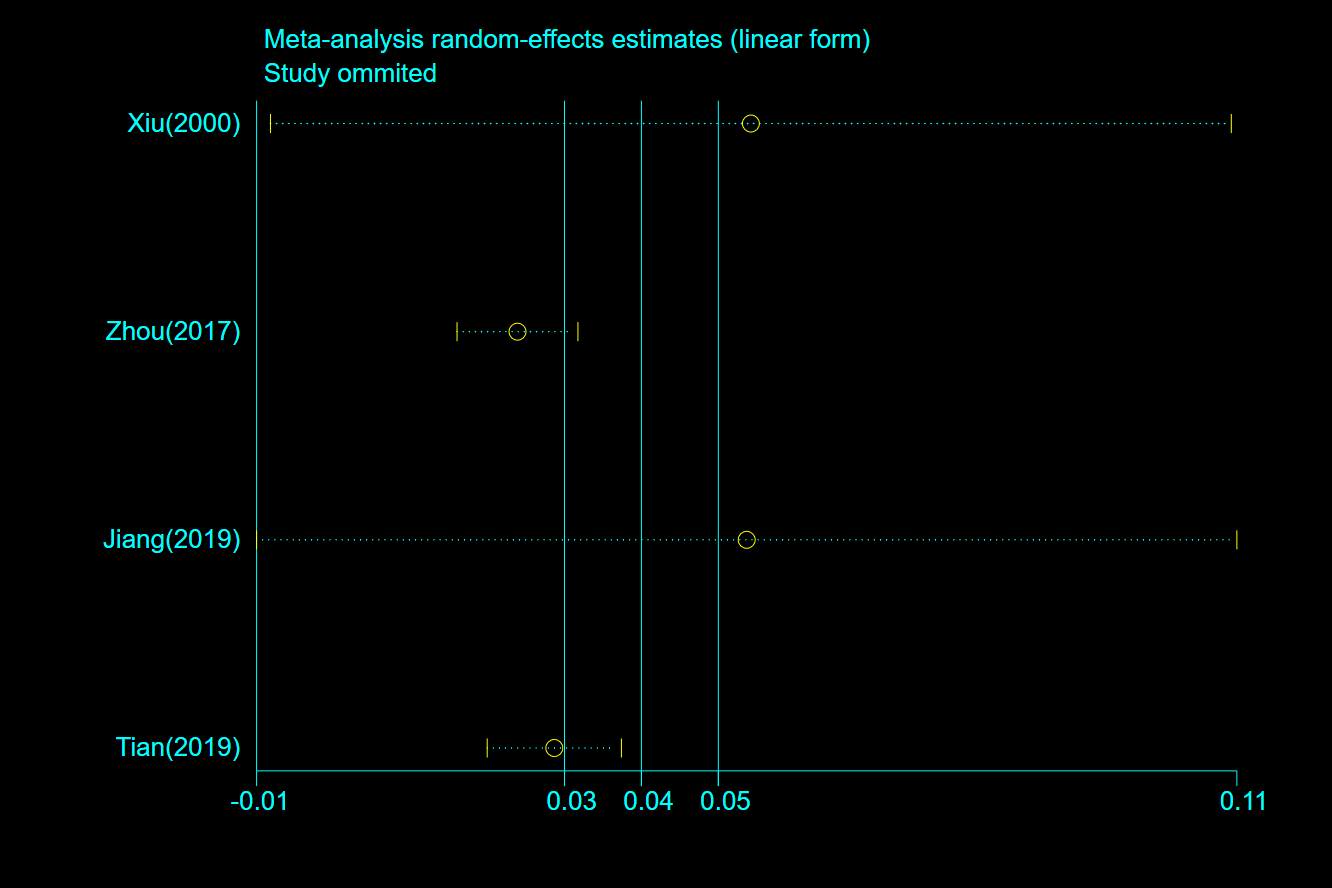


Figure 1 –Sensitivity analysis showing the prevalence of sepsis in hospital-wide


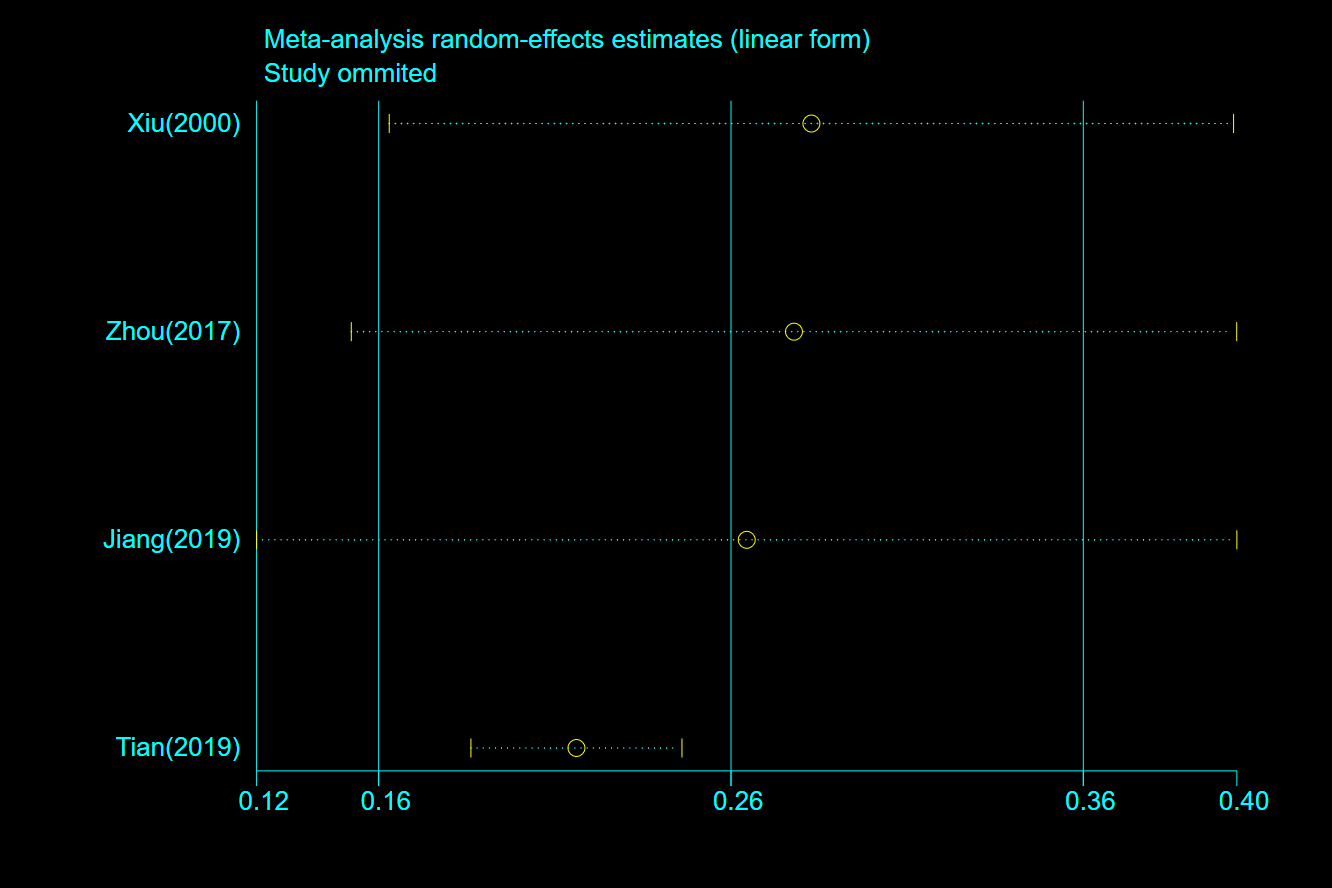


Figure 2 –Sensitivity analysis showing the mortality of sepsis in hospital-wide


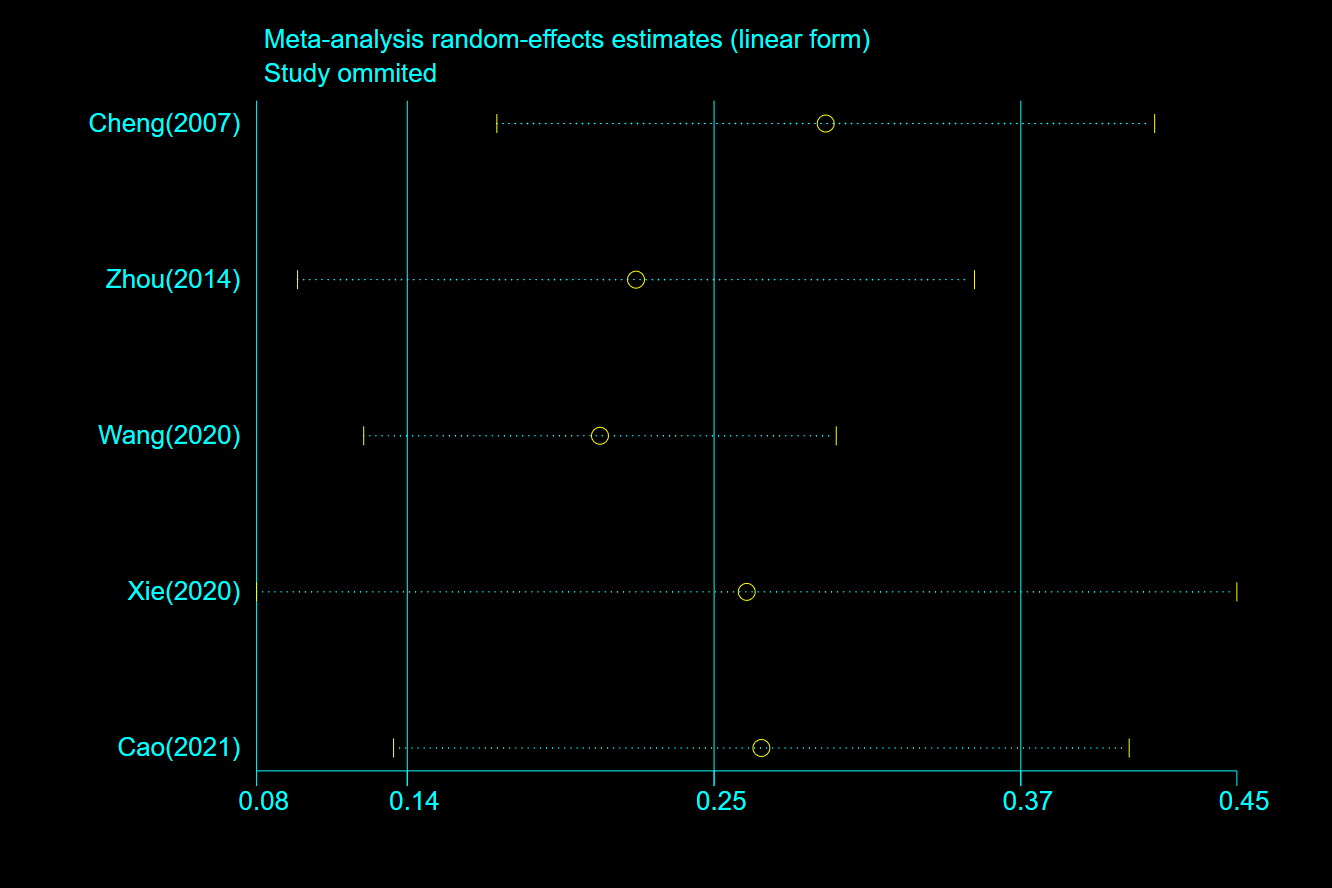


Figure 3 –Sensitivity analysis showing the prevalence of sepsis in ICUs


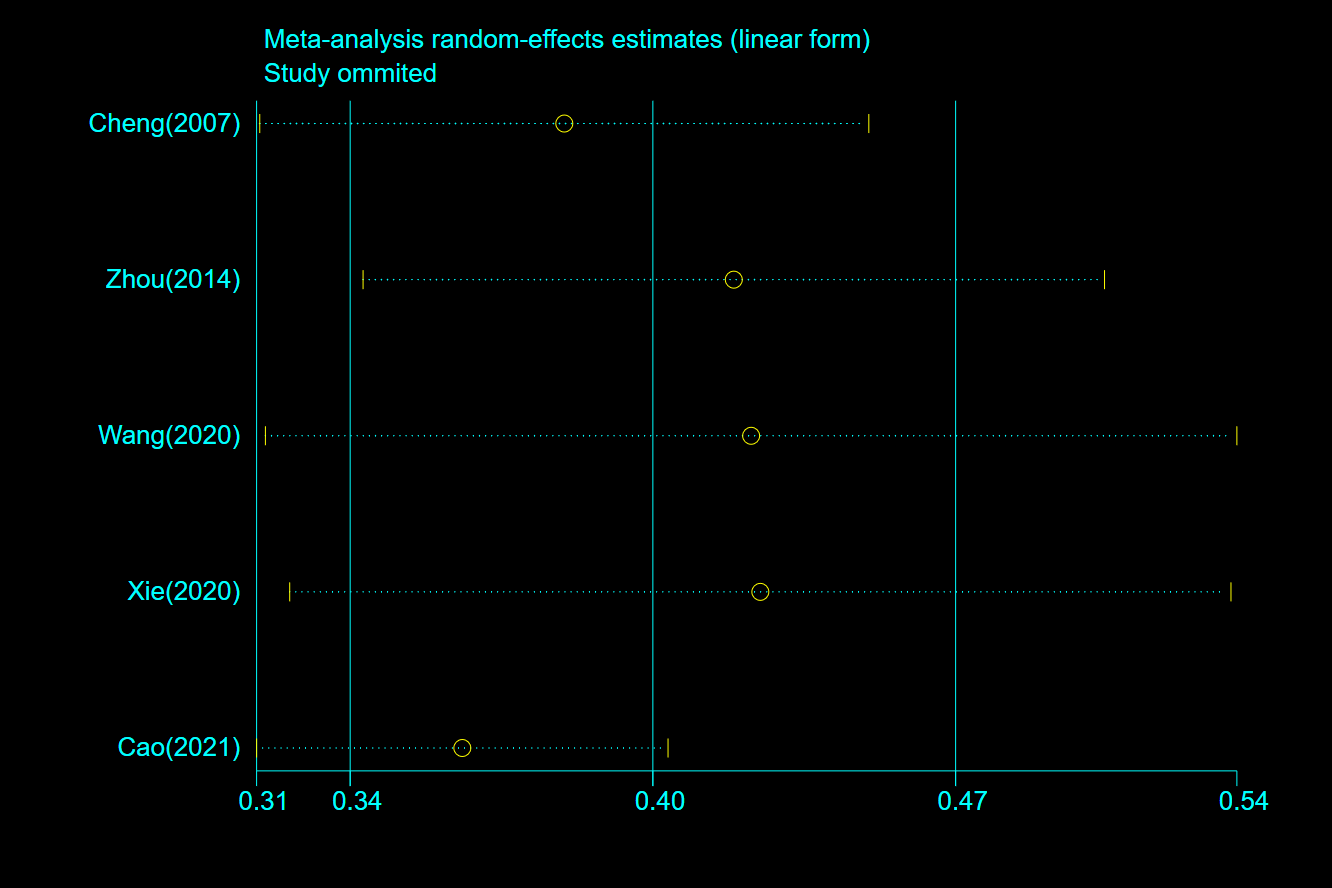


Figure 4 –Sensitivity analysis showing the mortality of sepsis in ICUs
